# Nonoscillatory Solutions for System of Neutral Dynamic Equations on Time Scales

**DOI:** 10.1155/2014/768215

**Published:** 2014-03-16

**Authors:** Zhanhe Chen, Taixiang Sun, Qi Wang, Hongjian Xi

**Affiliations:** ^1^College of Mathematics and Information Science, Guangxi University, Nanning, Guangxi 530004, China; ^2^College of Science, Guangxi University of Science and Technology, Liuzhou, Guangxi 545006, China; ^3^College of Information and Statistics, Guangxi University of Finance and Economics, Nanning, Guangxi 530003, China

## Abstract

We will discuss nonoscillatory solutions to the *n*-dimensional functional system of neutral type dynamic equations on time scales. We will establish some sufficient conditions for nonoscillatory solutions with the property lim_*t*→*∞*_ *⁡x*_*i*_(*t*) = 0, * i* = 1, 2,…, *n*.

## 1. Introduction

The theory of dynamic equations on time scales was introduced by its founder Hilger in his PHD thesis [1] in 1988. The study of dynamic equations on time scales is an area of mathematics that has recently received a lot of attention. It has been created in order to unify continuous and discrete analysis. In recent years there has been much research activity concerning the oscillation and nonoscillation of solutions of dynamic equations on time scales; we refer the reader to the papers [2–12]. In [13, 14], authors studied nonoscillatory solutions to the *n*-dimensional functional differential systems of neutral type and obtained some sufficient conditions for nonoscillatory solutions with the property lim⁡_*t*→*∞*_ *y*_*i*_(*t*) = 0, *i* = 1,2,…, *n*. Using the idea and method of [13, 14], in this paper, we will study the nonoscillatory solutions for systems of neutral dynamic equations on time scales, which have the following form:

(1) $$\begin{array}{c} {\left[x_{1}\left(t\right)-a\left(t\right)x_{1}\left(g\left(t\right)\right)\right]}^{\Delta }=p_{1}\left(t\right)x_{2}\left(t\right),\\ x_{i}^{\Delta }\left(t\right)=p_{i}\left(t\right)x_{i+1}\left(t\right),\;i=2,3,\ldots ,n-1,\\ x_{n}^{\Delta }\left(t\right)=\iota p_{n}\left(t\right)f\left(x_{1}\left(h\left(t\right)\right)\right),\;t\in 𝕋, \end{array}$$

where natural number *n* ≥ 3, |*ι* | = 1, *a* is a continuous, real-valued positive function defined on the time scale *𝕋* and through this paper we assume that

- *g* : *𝕋* → *𝕋* is a continuous and increasing function with lim⁡_*t*→*∞*_ *g*(*t*) = *∞* and {*t* ∈ *𝕋* : *t* ≥ *t*_0_} ⊂ *g*(*𝕋*) for some *t*_0_ ∈ *𝕋*;
- *p*_*i*_ : *𝕋* → ℝ^+^ : = [0, *∞*), *i* = 1,2,…, *n*, are continuous and increasing functions; *p*_*n*_ not identically equal to zero in any neighbourhood of infinity; ∫_*t*_^*∞*^ *p*_*k*_(*τ*)Δ*τ* = *∞*, *k* = 1,2,…, *n* − 1, hold for any *t* ∈ *𝕋*;
- *h* : *𝕋* → ℝ is a continuous and increasing function with lim⁡_*t*→*∞*_ *h*(*t*) = *∞*;
- *f* : ℝ → ℝ is a continuous function, and *f*(*u*)/*u* ≥ *K* for *u* ≠ 0, where *K* is a positive constant.

## 2. Some Preliminary Results

A time scale *𝕋* is an arbitrary nonempty closed subset of the real numbers. Throughout this paper, as a matter of convenience, for any *a*, *b* ∈ *𝕋*, *a* < *b*, we denote the sets {*t* ∈ *𝕋* : *a* ≤ *t* ≤ *b*} by [*a*, *b*]_*𝕋*_, which is called a close interval in *𝕋*. Open intervals and half-open intervals and so forth are defined accordingly.

A function *z*(*t*) is defined for *x*_1_(*t*) as

(2) $$\begin{array}{c} z\left(t\right)=x_{1}\left(t\right)-a\left(t\right)x_{1}\left(g\left(t\right)\right). \end{array}$$

A vector function *x* = (*x*_1_,…, *x*_*n*_) is a solution to the system (1) if there is a *t*_1_ ∈ *𝕋* such that functions *x*_*i*_(*t*), *i* = 1,2,…, *n*, are continuously differentiable and *x* satisfies (1) on [*t*_1_, *∞*)_*𝕋*_. Let *W* be the set of all solutions *x* = (*x*_1_,…, *x*_*n*_) to the system (1) satisfying sup⁡{∑_*i*=1_^*n*^|*x*_*i*_(*t*)| : *t* ∈ [*T*, *∞*)_*𝕋*_} > 0 for any *T* ∈ *𝕋*. A solution *x* ∈ *W* is called nonoscillatory if there exists a *T*_*x*_ ∈ *𝕋* such that every component is different from zero for *t* ∈ [*T*_*x*_, *∞*)_*𝕋*_. Otherwise a solution *x* ∈ *W* is said to be oscillatory.

Since we restrict our attention to asymptotic properties of nonoscillatory solutions to the system (1), we suppose that the time scale under consideration is not bounded above; that is, it is a time scale interval of the form [*t*_0_, +*∞*)_*𝕋*_. On any time scale we define the forward operator and delta derivative as follows.

**Definition (see [15])**

Let *𝕋* be a time scale. For any *t* ∈ *𝕋*, we define the forward jump operator *σ* : *𝕋* → *𝕋* by

(3) $$\begin{array}{c} \sigma \left(t\right):=\inf\left\{s\in 𝕋:s>t\right\}. \end{array}$$

**Definition 2 (see [15])**

Let *𝕋* be a time scale. Assume *f* : *𝕋* → ℝ is a function and let *t* ∈ *𝕋*. The (delta) derivative of *f* at *t* is defined by

(4) $$\begin{array}{c} f^{\Delta }\left(t\right)=\underset{s\to t}{\lim}\frac{f\left(\sigma \left(t\right)\right)-f\left(s\right)}{\sigma \left(t\right)-s},\;\text{where}\;s\in 𝕋\setminus \left\{\sigma \left(t\right)\right\}. \end{array}$$

For some other preliminary concepts on time scales, one can refer to [15]. In the remainder of this section, we present some lemmas indispensable which will be used later.

**Lemma 3**

Let *x* ∈ *W* be a solution to (1) with *x*_1_(*t*) ≠ 0 on [*t*_0_, *∞*)_*𝕋*_. Then *x* is nonoscillatory and *z*(*t*), *x*_2_(*t*),…, *x*_*n*_(*t*) are monotone on [*T*, *∞*)_*𝕋*_ for some *T* ∈ [*t*_0_, *∞*)_*𝕋*_.

**Proof**

Because *x*_1_(*t*) ≠ 0 on [*t*_0_, *∞*)_*𝕋*_, using the conditions (b) and (d), *x*_*n*_^Δ^(*t*) does not change its sign and not identically equal to zero on [*T*_1_, *∞*)_*𝕋*_ for some *T*_1_ ∈ [*t*_0_, *∞*)_*𝕋*_. This implies that *x*_*n*_(*t*) is monotone and *x*_*n*_(*t*) ≠ 0 on [*T*_2_, *∞*)_*𝕋*_ for some *T*_2_ ∈ [*T*_1_, *∞*)_*𝕋*_. Continue similarly as above and then conclude the desire results. The proof is complete.

**Lemma 4**

Assume that (a) holds and *g*(*t*) > *t* for *t* ∈ [*t*_0_, *∞*)_*𝕋*_. Let *y*(*t*) be a nonoscillatory solution to the functional inequality

(5) $$\begin{array}{c} y\left(t\right)\left[y\left(t\right)-a\left(t\right)y\left(g\left(t\right)\right)\right]>0,\;for\;any\;t\in {\left[t_{0},\infty \right)}_{𝕋}, \end{array}$$

where *a* : *𝕋* → [0, *∞*) is a continuous function. If 1 ≤ *a*(*t*) for *t* ∈ [*t*_0_, *∞*)_*𝕋*_, then *y*(*t*) is bounded on [*t*_0_, *∞*)_*𝕋*_. Moreover, if  1 < *λ* ≤ *a*(*t*) for *t* ∈ [*t*_0_, *∞*)_*𝕋*_, then lim⁡_*t*→*∞*_ *y*(*t*) = 0.

**Proof**

We firstly claim that there exists a negative integer *n*_*t*_ such that *t* = *g* ^*n*_*t*_^(*τ*) for some *τ* ∈ [*g* ^−1^(*t*_0_), *t*_0_)_*𝕋*_. In fact, if not the case, then there is *T* ∈ [*t*_0_, *∞*)_*𝕋*_ such that *g* ^−*n*^(*T*)∉[*g* ^−1^(*t*_0_), *t*_0_)_*𝕋*_ for any *n* ∈ *ℕ*. We need only to consider two cases: Case 1, *g* ^−*n*^(*T*)∈[*t*_0_, *T*)_*𝕋*_ for each *n* ∈ *ℕ*. Then there exists *b* ∈ [*t*_0_, *T*)_*𝕋*_ satisfying lim⁡_*n*→*∞*_ *g* ^−*n*^(*T*) = *b*, which implies *g*(*b*) = *b*, a contradiction. Case 2, *g* ^−(*n*+1)^(*T*) < *g* ^−1^(*t*_0_) < *t*_0_ < *g* ^−*n*^(*T*). Then, by the monotonicity of *g*, *g* ^−*n*^(*T*) = *g*(*g* ^−(*n*+1)^(*T*)) ≤ *g*(*g* ^−1^(*t*_0_)) = *t*_0_, also a contradiction.

Without loss of generality, we may assume that *y*(*t*) is a positive solution of the functional inequality *y*(*t*)[*y*(*t*) − *a*(*t*)*y*(*g*(*t*))] > 0 on [*t*_0_, *∞*)_*𝕋*_. Then *y*(*t*) > *a*(*t*)*y*(*g*(*t*)) for all *t* ∈ [*t*_0_, *∞*)_*𝕋*_.

If 1 ≤ *a*(*t*) for *t* ∈ [*t*_0_, *∞*)_*𝕋*_, then *y*(*t*) > *y*(*g*(*t*)) ≥ *y*(*g* ^*n*^(*t*)), for any *t* ∈ [*t*_0_, *T*)_*𝕋*_ and each *n* ∈ *ℕ*. For any *t* ∈ [*t*_0_, *∞*)_*𝕋*_, from the claim above, let *t* = *g* ^*n*_*t*_^(*τ*_*t*_) for some *τ*_*t*_ ∈ [*g* ^−1^(*t*_0_), *t*_0_)_*𝕋*_ and some *n*_*t*_ ∈ *ℕ*. Then *y*(*t*) = *y*(*g* ^*n*_*t*_^(*τ*_*t*_)) ≤ *y*(*τ*_*t*_) ≤ *M* : = max⁡{*y*(*s*) | *s* ∈ [*g* ^−1^(*t*_0_), *t*_0_)_*𝕋*_}. Therefore, *y*(*t*) is bounded on [*t*_0_, *∞*)_*𝕋*_.

If 1 < *λ* ≤ *a*(*t*) for *t* ∈ [*t*_0_, *∞*)_*𝕋*_, then *y*(*t*) > *λy*(*g*(*t*)) ≥ *λ* ^*n*^ *y*(*g* ^*n*^(*t*)) for any *t* ∈ [*t*_0_, *∞*)_*𝕋*_ and each *n* ∈ *ℕ*. For any *t* ∈ [*t*_0_, *∞*)_*𝕋*_, from the claim above, let *t* = *g* ^*n*_*t*_^(*τ*_*t*_) for some *τ*_*t*_ ∈ [*g* ^−1^(*t*_0_), *t*_0_)_*𝕋*_ and some *n*_*t*_ ∈ *ℕ*. Then 0 < *y*(*t*)≤(1/*λ* ^*n*^)*y*(*τ*_*t*_)≤(1/*λ* ^*n*^)*M*, which implies that lim⁡_*t*→*∞*_ *y*(*t*) = 0.

Similarly as Lemma 4, we can prove the following lemma.

**Lemma 5**

Assume that (a) holds and *g*(*t*) < *t* for *t* ∈ [*t*_0_, *∞*)_*𝕋*_. Let *y*(*t*) be a nonoscillatory solution to the functional inequality:

(6) $$\begin{array}{c} y\left(t\right)\left[y\left(t\right)-a\left(t\right)y\left(g\left(t\right)\right)\right]<0,\;for\;any\;t\in {\left[t_{0},\infty \right)}_{𝕋}, \end{array}$$

where *a* : *𝕋* → [0, *∞*) is a continuous function. If 0 < *a*(*t*) ≤ 1 for *t* ∈ [*t*_0_, *∞*)_*𝕋*_, then *y*(*t*) is bounded on [*t*_0_, *∞*)_*𝕋*_. Moreover, if 0 < *a*(*t*) ≤ *λ* < 1 for *t* ∈ [*t*_0_, *∞*)_*𝕋*_, then lim⁡_*t*→*∞*_ *y*(*t*) = 0.

**Lemma 6**

Assume that *q* : *𝕋* → ℝ^+^ and *δ* : *𝕋* → *𝕋* are continuous functions and *δ*(*t*) > *σ*(*t*) for all *t* ∈ *𝕋*. If lim⁡⁡inf⁡_*t*→*∞*_∫_*σ*(*t*)_^*δ*(*t*)^ *q*(*s*)Δ*s* > 1/*e*, then the following two statements hold.

(i) The functional inequality
  (7) $$\begin{array}{c} x^{\Delta }\left(t\right)-q\left(t\right)x\left(\delta \left(t\right)\right)\geq 0,\;t\in 𝕋, \end{array}$$
  has no eventually positive solution.
(ii) The functional inequality
  (8) $$\begin{array}{c} x^{\Delta }\left(t\right)-q\left(t\right)x\left(\delta \left(t\right)\right)\leq 0,\;t\in 𝕋, \end{array}$$
  has no eventually negative solution.

**Proof**

We only prove (i) for the proof of (ii) is similar. Suppose that the functional inequality (7) has an eventually positive solution *x*(*t*). Without loss of generality, we may assume that *x*(*t*) > 0 for any *t* ∈ *𝕋*. Then, from (7), *x* ^Δ^(*t*) ≥ *q*(*t*)*x*(*δ*(*t*)) ≥ 0 for any *t* ∈ *𝕋*. It follows that *x*(*t*) is nondecreasing on *𝕋*.

Let *X* : ℝ → ℝ be the linear extension of the function *x*(*t*), then *X* : ℝ → ℝ is continuous, *X* : *𝕋* → ℝ is delta differentiable, and *X* ^Δ^ = *x* ^Δ^ on *𝕋* ^*κ*^. By [15, Theorem 1.87], for any *t* ∈ *𝕋* ^*κ*^, there is *ξ*_*t*_ ∈ [*t*, *σ*(*t*)] such that [ln⁡*x*(*t*)]^Δ^ = *x* ^Δ^(*t*)/*X*(*ξ*_*t*_). Because *δ*(*t*) > *σ*(*t*) ≥ *ξ*_*t*_ ≥ *t* for all *t* ∈ *𝕋* ^*κ*^, *x*(*δ*(*t*)) ≥ *x*(*σ*(*t*)) ≥ *X*(*ξ*_*t*_) ≥ *x*(*t*) > 0 for all *t* ∈ *𝕋* ^*κ*^. Therefore,

(9) $$\begin{array}{c} {\left[\ln x\left(t\right)\right]}^{\Delta }\geq \frac{x^{\Delta }\left(t\right)}{x\left(\sigma \left(t\right)\right)}\geq \frac{x^{\Delta }\left(t\right)}{x\left(\delta \left(t\right)\right)},\;\text{for}\;t\in {𝕋}^{\kappa }. \end{array}$$

By (7), we have

(10) $$\begin{array}{c} {\left[\ln x\left(t\right)\right]}^{\Delta }\geq q\left(t\right),\;\text{for}\;t\in {𝕋}^{\kappa }. \end{array}$$

Integrating the inequality above from *σ*(*t*) to *δ*(*t*) on *𝕋*, we get, for *t* ∈ *𝕋* ^*κ*^,

(11) $$\begin{array}{c} x\left(\delta \left(t\right)\right)\geq x\left(\sigma \left(t\right)\right)\exp\left[\int_{\sigma (t)}^{\delta (t)}q\left(s\right)\Delta s\right]. \end{array}$$

By the hypothesis, there exist *t*_1_ ∈ *𝕋* and a constant *c* such that ∫_*σ*(*t*)_^*δ*(*t*)^ *q*(*s*)Δ*s* ≥ *c* > 1/*e* holds for *t* ∈ [*t*_1_, *∞*)_*𝕋*_. So we have for *t* ∈ [*t*_1_, *∞*)_*𝕋*_,

(12) $$\begin{array}{c} x\left(\delta \left(t\right)\right)\geq e^{c}x\left(\sigma \left(t\right)\right)\geq ec\cdot x\left(\sigma \left(t\right)\right). \end{array}$$

Here, to give the last inequality, we have used the inequality *e* ^*y*^ > *ey* for all *y* ≥ 0. From (7) and (9), we have

(13) $$\begin{array}{c} {\left[\ln x\left(t\right)\right]}^{\Delta }\geq ec\cdot q\left(t\right),\;\text{for}\;t\in {\left[t_{1},\infty \right)}_{𝕋}. \end{array}$$

Integrating the inequality above from *σ*(*t*) to *δ*(*t*) on *𝕋*, we get, for *t* ∈ [*t*_1_, *∞*)_*𝕋*_,

(14) x(δ(t))≥x(σ(t))exp⁡⁡[∫σ(t)δ(t)ec·q(s)Δs]≥eec2x(σ(t))≥(ec)2·x(σ(t)).

Continuing this progress, we conclude that, for each natural number *n*,

(15) $$\begin{array}{c} x\left(\delta \left(t\right)\right)\geq {\left(ec\right)}^{n}x\left(\sigma \left(t\right)\right),\;t\in {\left[t_{1},\infty \right)}_{𝕋}. \end{array}$$

Since *ec* > 1, it follows that *x*(*δ*(*t*)) = *∞* for any *t* ∈ [*t*_1_, *∞*)_*𝕋*_, a contradiction. The proof is complete.

Let *x*(*t*) ∈ *W* be a nonoscillatory solution to (1). It follows, from Lemma 3, that the function *z*(*t*) has to be eventually of constant sign. Hence, either

(16) $$\begin{array}{c} x_{1}\left(t\right)z\left(t\right)>0 \end{array}$$

or

(17) $$\begin{array}{c} x_{1}\left(t\right)z\left(t\right)<0 \end{array}$$

for sufficiently large *t* ∈ *𝕋*.

**Lemma**

Let *x*(*t*) be a nonoscillatory solution to (1) on [*t*_0_, *∞*)_*𝕋*_, and *x*_1_(*t*)*z*(*t*) > 0 for *t* ∈ [*t*_0_, *∞*)_*𝕋*_. Then there exist *l* ∈ {1,2,…, *n*} with *ι* · (−1)^*n*+*l*+1^ = 1 or *l* = *n* and some *t*_1_ ∈ [*t*_0_, *∞*)_*𝕋*_, such that for *t* ∈ [*t*_1_, *∞*)_*𝕋*_

(18) $$\begin{array}{c} x_{i}\left(t\right)z\left(t\right)>0,\;i=1,2,\ldots ,l,\\ {\left(-1\right)}^{i+l}x_{i}\left(t\right)z\left(t\right)>0,\;i=l+1,\ldots ,n. \end{array}$$

**Proof**

Putting *A* = {*k* ∈ {1,2,…, *n*} : *x*_*i*_(*t*)*z*(*t*) > 0 for *t* ∈ [*t*_0_, *∞*)_*𝕋*_ and *i* = 1,2,…, *k*, then *A* ≠ *∅* and so we can pick *l* = max⁡{*k* : *k* ∈ *A*}. It is obvious that 1 ≤ *l* ≤ *n*. Without loss of generality, we may assume that *x*_1_(*t*) > 0 for *t* ∈ [*t*_0_, *∞*)_*𝕋*_. Note that *z*(*t*) > 0 for all *t* ∈ [*t*_0_, *∞*)_*𝕋*_. If *l* = *n*, it is obvious that (18) holds. Next, we assume 1 ≤ *l* < *n*, then *x*_*l*_(*t*) > 0, *x*_*l*+1_(*t*) < 0 for *t* ∈ [*t*_0_, *∞*)_*𝕋*_. We will show recursively that (18) holds.

We firstly show that *x*_*l*+2_(*t*) > 0 for *t* ∈ [*t*_0_, *∞*)_*𝕋*_. Otherwise, *x*_*l*+2_(*t*) < 0 for *t* ∈ [*t*_0_, *∞*)_*𝕋*_. Then, from (1) and (b), we have *x*_*l*+1_(*t*) ≤ *x*_*l*+1_(*t*_0_) < 0 for *t* ∈ [*t*_0_, *∞*)_*𝕋*_. So, by (1) and (b),

(19) xl(t0)>−xl(t)+xl(t0)=−∫t0tpl(s)xl+1(s)Δs≥−xl+1(t0)∫t0tpl(s)Δs

holds for all *t* ∈ [*t*_0_, *∞*)_*𝕋*_, which contradicts the condition (b). Hence, *x*_*l*+1_(*t*) < 0, *x*_*l*+2_(*t*) > 0 for *t* ∈ [*t*_0_, *∞*)_*𝕋*_.

We now show that *x*_*l*+3_(*t*) < 0 for *t* ∈ [*t*_0_, *∞*)_*𝕋*_. Otherwise, *x*_*l*+3_(*t*) > 0 for *t* ∈ [*t*_0_, *∞*)_*𝕋*_. Then, from (1) and (b), we have *x*_*l*+2_(*t*) ≥ *x*_*l*+2_(*t*_0_) > 0 for *t* ∈ [*t*_0_, *∞*)_*𝕋*_. So, by (1) and (b),

(20) $$\begin{array}{c} x_{l+1}\left(t_{0}\right)<-\int_{t_{0}}^{t}p_{l+1}\left(s\right)x_{l+2}\left(s\right)\Delta s\leq -x_{l+2}\left(t_{0}\right)\int_{t_{0}}^{t}p_{l+1}\left(s\right)\Delta s \end{array}$$

holds for all *t* ∈ [*t*_0_, *∞*)_*𝕋*_, which contradicts the condition (b). This implies that *x*_*l*+2_(*t*) > 0, *x*_*l*+3_(*t*) < 0 for *t* ∈ [*t*_0_, *∞*)_*𝕋*_.

Continuing this progress, we can prove that (18) holds. To complete the proof, it remains to show that *ι* · (−1)^*n*+*l*+1^ = 1. Suppose *ι* · (−1)^*n*+*l*^ = 1, then, from (1) and (d), we have (−1)^*n*+*l*+1^ *y*_*n*_^Δ^(*t*) < 0 on [*t*_2_, *∞*)_*𝕋*_ for $t_{2}\in {\left[{\tilde{t}}_{1},\infty \right)}_{𝕋}$. Note that (−1)^*n*−1+*l*^ *x*_*n*−1_(*t*) > 0 and (−1)^*n*+*l*^ *x*_*n*_(*t*) > 0 for all *t* ∈ [*t*_2_, *∞*)_*𝕋*_. It is easy to show that |*x*_*n*_(*t*)|≥|*x*_*n*_(*t*_2_)|>0 for all *t* ∈ [*t*_2_, *∞*)_*𝕋*_. But then,

(21) |xn−1(t2)|>|∫t2tpn−1(s)xn(s)Δs|=∫t2tpn−1(s)|xn(s)|Δs≥|xn(t2)|∫t2tpn−1(s)Δs

holds for all *t* ∈ [*t*_2_, *∞*)_*𝕋*_, which contradicts the condition (b). This completes the proof.

**Lemma**

Let *x*(*t*) be a nonoscillatory solution to (1) on [*t*_0_, *∞*)_*𝕋*_, and *x*_1_(*t*)*z*(*t*) < 0 for *t* ∈ [*t*_0_, *∞*)_*𝕋*_. Then there exist *l* ∈ {1,2,…, *n*} with *ι* · (−1)^*n*+*l*^ = 1 or *l* = *n* and some *t*_1_ ∈ [*t*_0_, *∞*)_*𝕋*_, such that for *t* ∈ [*t*_1_, *∞*)_*𝕋*_, either

(22) $$\begin{array}{c} x_{1}\left(t\right)z\left(t\right)<0,\;\;{\left(-1\right)}^{i}x_{i}\left(t\right)z\left(t\right)>0,\;i=2,3,\ldots ,n \end{array}$$

or

(23) $$\begin{array}{c} x_{1}\left(t\right)z\left(t\right)<0,\;\;x_{i}\left(t\right)z\left(t\right)>0,\;i=2,3,\ldots ,l,\\ {\left(-1\right)}^{i+l}x_{i}\left(t\right)z\left(t\right)>0,\;i=l+1,\ldots ,n. \end{array}$$

**Proof**

Without loss of generality, we may assume that *x*_1_(*t*) > 0 for *t* ∈ [*t*_0_, *∞*)_*𝕋*_. Note that *z*(*t*) < 0 for all *t* ∈ [*t*_0_, *∞*)_*𝕋*_. We consider the following two cases.

*(I) x*_2_(*t*) > 0* for t* ∈ [*t*_0_, *∞*)_*𝕋*_. We will show recursively that (22) holds. We firstly show that *x*_3_(*t*) < 0 for *t* ∈ [*t*_0_, *∞*)_*𝕋*_. Otherwise, *x*_3_(*t*) > 0 for *t* ∈ [*t*_0_, *∞*)_*𝕋*_. Then, from (1) and (b), it is easy to see that *x*_2_(*t*) ≤ *x*_2_(*t*_1_) < 0 for *t* ∈ [*t*_0_, *∞*)_*𝕋*_. So, by (1) and (b) again,

(24) z(t0)<−z(t)+z(t0)  =−∫t0tpl(s)x2(s)Δs≤−x2(t0)∫t0tpl(s)Δs

holds for all *t* ∈ [*t*_0_, *∞*)_*𝕋*_, which contradicts the condition (b). Hence, *x*_2_(*t*) > 0 and *x*_3_(*t*) < 0 for *t* ∈ [*t*_0_, *∞*)_*𝕋*_. Continuing this progress similarly, we conclude that (22) holds.

*(II) x*_2_(*t*) < 0* for t* ∈ [*t*_0_, *∞*)_*𝕋*_. Analogically as in the proof of Lemma 7, we can prove that (23) holds. This completes the proof.

For the sake of convenience, we denote by *N*_*l*_^+^, *N*_1_^−^, and *N*_*l*_^−^ the set of all nonoscillatory solutions to the system (1) satisfying (18), (22), and (23) correspondingly. Denote by *N* the set of all nonoscillatory solutions to the system (1). From Lemmas 7 and 8, we have the classification of nonoscillatory solutions to the system (1) as follows:

(i) *n* is odd and *ι* = 1,
  (25) $$\begin{array}{c} N=N_{2}^{+}\cup N_{4}^{+}\cup \cdots \cup N_{n-1}^{+}\cup N_{n}^{+}\cup N_{1}^{-}\cup N_{3}^{-}\cup \cdots \cup N_{n}^{-}; \end{array}$$
(ii) *n* is odd and *ι* = −1,
  (26) $$\begin{array}{c} N=N_{1}^{+}\cup N_{3}^{+}\cup \cdots \cup N_{n}^{+}\cup N_{2}^{-}\cup N_{4}^{-}\cup \cdots \cup N_{n-1}^{-}\cup N_{n}^{-}; \end{array}$$
(iii) *n* is even and *ι* = 1,
  (27) $$\begin{array}{c} N=N_{1}^{+}\cup N_{3}^{+}\cup \cdots \cup N_{n-1}^{+}\cup N_{n}^{+}\cup N_{2}^{-}\cup N_{4}^{-}\cup \cdots \cup N_{n}^{-}; \end{array}$$
(iv) *n* is even and *ι* = −1,
  (28) $$\begin{array}{c} N=N_{2}^{+}\cup N_{4}^{+}\cup \cdots \cup N_{n}^{+}\cup N_{1}^{-}\cup N_{3}^{-}\cup \cdots \cup N_{n-1}^{-}\cup N_{n}^{-}. \end{array}$$

**Remark 9**

Assume that *g*(*t*) < *t* and 0 < *a*(*t*) ≤ *λ* < 1 for *t* ∈ [*t*_0_, *∞*)_*𝕋*_, where *λ* is a constant. Then *N*_*k*_^−^ = *∅* for *k* ∈ {2,3,…, *n*}.

In fact, if *N*_*k*_^−^ ≠ *∅* for some *k* ∈ {2,3,…, *n*}. Let *x* ∈ *N*_*k*_^−^ and suppose, without loss of generality, that *x*_1_(*t*) > 0 for *t* ∈ [*t*_0_, *∞*)_*𝕋*_. Note that *z*(*t*) < 0 and *x*_2_(*t*) > 0 for *t* ∈ [*t*_0_, *∞*)_*𝕋*_. It follows that *z*(*t*) ≤ *z*(*t*_1_) < 0 for *t* ∈ [*t*_0_, *∞*)_*𝕋*_. On the other hand, 0 > *z*(*t*)≥−*a*(*t*)*x*_1_(*g*(*t*))≥−*λx*_1_(*g*(*t*)) for *t* ∈ [*t*_0_, *∞*)_*𝕋*_. By Lemma 5, lim⁡_*t*→*∞*_ *x*_1_(*t*) = 0 which implies that lim⁡_*t*→*∞*_ *z*(*t*) = 0, a contradiction.

**Lemma 10**

Let *x*(*t*) be a nonoscillatory solution to (1) on [*t*_0_, *∞*)_*𝕋*_. Suppose that lim⁡_*t*→*∞*_ | *z*(*t*)| = *L*_1_, and lim⁡_*t*→*∞*_ | *x*_*k*_(*t*)| = *L*_*k*_ for *k* ∈ {2,3,…, *n*}. Then

(29) Lk>0, k∈{2,3,…,n}  implies  Li=∞,i=1,2,…, k−1,

(30) Lk<∞, k∈{1,2,…,n−1}  implies  Li=0,i=k+1,…,n.

**Proof**

Suppose that *L*_*k*_ > 0, *k* ∈ {2,3,…, *n*}, then there is a positive constant *M*_*k*_ such that |*x*_*k*_(*t*)|≥*M*_*k*_ for *t* ∈ [*t*_0_, *∞*)_*𝕋*_. By (1) and (b), we have

(31) |xk−1(t)|>|∫t0tpk−1(s)xk(s)Δs|≥∫t0tpk−1(s)|xk(s)|Δs≥Mk∫t0tpk−1(s)Δs.

It follows from (b) that *L*_*k*−1_ = *∞*. Obviously there is a positive constant *M*_*k*−1_ such that |*x*_*k*−1_(*t*)|≥*M*_*k*−1_ for *t* ∈ [*t*_0_, *∞*)_*𝕋*_. Similarly as above, we can prove that *L*_*k*−2_ = *∞*. Continuing this progress, we conclude that (29) holds. Using (29), it is easy to see that (30) holds. The proof is complete.

## 3. Main Results and Proofs

We will now give the main results and their proofs. In the squeal, for the convenience of expressions, we define *I*_*k*_ and *J*_*k*_ by recursion formula as follows:

(32) I0(s,t)≡1,Ik(s,t;h1,h2,…,hk)=∫tsh1(x)Ik−1(x,t;h2,h3,…,hk)Δx,J0(s,t)≡1,Jk(s,t;h1,h2,…,hk)  =∫tshk(x)Jk−1(s,x;h1,h2,…,hk−1)Δx,

where *h*_*k*_ : *𝕋* → ℝ, *k* = 1,2,…, *n*, are continuous functions and *s*, *t* ∈ *𝕋*.

**Remark 11**

From the definitions of *I*_*k*_ and *J*_*k*_, it is easy to show the following two properties:

(i) if *h*_*i*_ ≥ 0 for each *i* = 1,2,…, *k*, then
  (33) $$\begin{array}{c} I_{k}\left(s,t;h_{1},h_{2},\ldots ,h_{k}\right)\geq I_{k}\left(u,v;h_{1},h_{2},\ldots ,h_{k}\right)\geq 0 \end{array}$$
  for *s* ≥ *u* ≥ *v* ≥ *t*, *s*, *u*, *v*, *t* ∈ *𝕋*;
(ii) if *h*_*i*_ ≥ 0 for each *i* = 1,2,…, *k* and *h*_*l*_ ≥ *f*_*l*_ for some *l* ∈ {1,2,…, *k*}, then
  (34) Ik(s,t;h1,h2,…,hk)  ≥Ik(s,t;h1,h2,…,hl−1,fl,hl+1,…,hk)
  for *s* > *t*, *s*, *t* ∈ *𝕋*.

**Theorem 12**

Assume that *n* is odd and *ι* = −1. If the following statements hold:

(1) there is a constant *λ* such that, for any *t* ∈ [*t*_0_, *∞*)_*𝕋*_,
  (35) $$\begin{array}{c} 1<\lambda \leq a\left(t\right); \end{array}$$
(2) for any *t* ∈ [*t*_0_, *∞*)_*𝕋*_,
  (36) $$\begin{array}{c} \sigma \left(t\right)<g\left(t\right)<h\left(t\right); \end{array}$$
(3) there is a continuous function *α* : [*t*_0_, *∞*)_*𝕋*_ → [*t*_0_, *∞*)_*𝕋*_ such that
  (37) $$\begin{array}{c} t<\alpha \left(t\right), \end{array}$$
  (38) liminf⁡t→∞∫σ(t)g−1(h(t))Kp1(v)Jn−1×(α(v),v;pna(g−1(h)),pn−1,…,p2)Δv>1e;
(4) for each even *l* with 4 ≤ *l* ≤ *n*,
  (39) lim⁡ sup⁡t→∞ KIl−1(t,h−1(g(t));p1,…,pl−2,pl−1(∗)×Jn−l+1(t,(∗);pna(g−1(h)),pn−1,…,pl))  >1;
(5) (40) $$\begin{array}{c} \underset{t\to \infty }{\mathrm{limsup}}\;KI_{n}\left(t,h^{-1}\left(g\left(t\right)\right);p_{1},p_{2},\ldots ,p_{n-1},\frac{p_{n}}{a\left(g^{-1}\left(h\right)\right)}\right)>1, \end{array}$$

then, for every nonoscillatory solution *x* ∈ *W* to (1), lim⁡_*t*→*∞*_ *x*_*i*_(*t*) = 0, *i* = 1,2,…, *n*.

**Proof**

Let *x* ∈ *W* be a nonoscillatory solution to (1). Without loss of generality, we may assume that *x*_1_(*t*) > 0 for all *t* ∈ [*t*_1_, *∞*)_*𝕋*_ ⊂ [*t*_0_, *∞*)_*𝕋*_. Because *n* is odd and *ι* = −1, the expression (26) holds. We consider the following five cases.

*(I) x* ∈ *N*_1_^+^ *on *[*t*_1_, *∞*)_*𝕋*_. In this case, we have

(41) x1(t)>0,  z(t)>0,  x2(t)<0,  x3(t)>0,…,xn(t)>0,t∈[t1,∞)𝕋.

By Lemma 4, lim⁡_*t*→*∞*_ *x*_1_(*t*) = 0. From (1) and (41), we have *z*(*t*) is a positive and nonincreasing function, which implies that lim⁡_*t*→*∞*_ | *z*(*t*)| = *L*_1_ < *∞*. It follows, from Lemma 10, that lim⁡_*t*→*∞*_ *x*_*i*_(*t*) = 0, *i* = 1,2,…, *n*.

*(II) x* ∈ *N*_3_^+^ ∪ *N*_5_^+^ ∪ ⋯∪*N*_*n*_^+^ *on *[*t*_1_, *∞*)_*𝕋*_. In this case, we have

(42) x1(t)>0,  z(t)>0,  x2(t)>0,  x3(t)>0, t∈[t1,∞)𝕋.

From (1) and (42), there is a positive constant *M* such that *x*_2_(*t*) ≥ *M* for *t* ∈ [*t*_1_, *∞*)_*𝕋*_. Integrating the first equation of (1) over [*t*_1_, *t*]_*𝕋*_, and using the inequality above, we get

(43) $$\begin{array}{c} z\left(t\right)\geq z\left(t_{1}\right)+M\int_{t_{1}}^{t}p_{1}\left(\tau \right)\Delta \tau ,\;t\in {\left[t_{1},\infty \right)}_{𝕋}. \end{array}$$

By the condition (b), lim⁡_*t*→*∞*_ *z*(*t*) = *∞*. The conditions (35), (36), and (42) imply that *x*_1_(*t*) is bounded on [*t*_1_, *∞*)_*𝕋*_, which arrives a contradiction as *z*(*t*) < *x*_1_(*t*) for *t* ∈ [*t*_1_, *∞*)_*𝕋*_. Therefore, *N*_3_^+^ ∪ *N*_5_^+^ ∪ ⋯∪*N*_*n*_^+^ = *∅*.

*(III) x* ∈ *N*_2_^−^ *on *[*t*_1_, *∞*)_*𝕋*_. In this case, we have

(44) x1(t)>0,  z(t)<0,  x2(t)<0,  x3(t)>0,…,xn(t)>0,t∈[t1,∞)𝕋.

Integrating the second equation of (1) from *t* to *s*, we obtain that, for *s* ≥ *t* and *s*, *t* ∈ [*t*_1_, *∞*)_*𝕋*_,

(45) $$\begin{array}{c} x_{2}\left(t\right)\leq -\int_{t}^{s}p_{2}\left(u_{2}\right)x_{3}\left(u_{2}\right)\Delta u_{2}. \end{array}$$

Integrating the third equation of (1) from *u*_2_ to *s*, we get that, for *s* ≥ *u*_2_ ≥ *t* and *s*, *u*_2_, *t* ∈ [*t*_1_, *∞*)_*𝕋*_,

(46) $$\begin{array}{c} -x_{3}\left(u_{2}\right)\leq \int_{u_{2}}^{s}p_{3}\left(u_{3}\right)x_{4}\left(u_{3}\right)\Delta u_{3}. \end{array}$$

Continuing this progress, we have that, for any *s* ≥ *u*_*n*−1_ ≥ *u*_*n*−2_ ≥ ⋯≥*u*_2_ ≥ *t* and *s*, *u*_*i*_, *t* ∈ [*t*_1_, *∞*)_*𝕋*_, 2 ≤ *i* ≤ *n* − 1,

(47) (−1)kxk(uk−1)≤(−1)k+1∫uk−1spk(uk)xk+1(uk)Δuk,3≤k≤k−1,−xn(un−1)≤∫un−1sxnΔ(un)Δun.

Combining *n* − 1 inequalities above, we obtain that, for *s* ≥ *t* and *s*, *t* ∈ [*t*_1_, *∞*)_*𝕋*_,

(48) x2(t)≤∫tsp2(u2)∫u2sp3(u3)⋯∫un−2spn−1(un−1) ×∫un−1sxnΔ(un)Δun⋯Δu2=Jn−1(s,t;xnΔ,pn−1,…,p2).

From (1), (b), (d), and (44), we have that, for *s* ≥ *u*_*n*_ ≥ *t* and *s*, *u*_*n*_, *t* ∈ [*t*_1_, *∞*)_*𝕋*_,

(49) $$\begin{array}{c} x_{n}^{\Delta }\left(u_{n}\right)\leq -Kp_{n}\left(u_{n}\right)x_{1}\left(h\left(u_{n}\right)\right). \end{array}$$

Substituting (49) to (48), we have that, for *s* ≥ *t* and *s*, *t* ∈ [*t*_1_, *∞*)_*𝕋*_,

(50) $$\begin{array}{c} x_{2}\left(t\right)\leq KJ_{n-1}\left(s,t;-p_{n}x_{1}\left(h\right),p_{n-1},\ldots ,p_{2}\right). \end{array}$$

Because of *x*_1_(*t*) > 0 for *t* ∈ [*t*_1_, *∞*)_*𝕋*_,

(51) $$\begin{array}{c} -x_{1}\left(h\left(t\right)\right)<\frac{z\left(g^{-1}\left(h\left(t\right)\right)\right)}{a\left(g^{-1}\left(h\left(t\right)\right)\right)},\;t\in {\left[t_{1},\infty \right)}_{𝕋}. \end{array}$$

Using the monotonicity of *z*(*g* ^−1^(*h*(*t*))), we have that, for *s* ≥ *u*_*n*_ ≥ *t* and *s*, *u*_*n*_, *t* ∈ [*t*_1_, *∞*)_*𝕋*_,

(52) $$\begin{array}{c} -x_{1}\left(h\left(u_{n}\right)\right)\leq \frac{z\left(g^{-1}\left(h\left(t\right)\right)\right)}{a\left(g^{-1}\left(h\left(u_{n}\right)\right)\right)}. \end{array}$$

Combining (50) and (52), we have that, for *s* ≥ *t* and *s*, *t* ∈ [*t*_1_, *∞*)_*𝕋*_,

(53) x2(t) ≤[KJn−1(s,t;pna(g−1(h)),pn−1,…,p2)]z(g−1(h(t))).

Multiplying the inequality above by *p*_1_(*t*) and putting *s* = *α*(*t*), we get that

(54) zΔ(t)−[Kp1(t)Jn−1(α(t),t;pna(g−1(h)),pn−1,…,p2)]  ×z(g−1(h(t)))≤0, t∈[t1,∞)𝕋.

By Lemma 6, the inequality above has no eventually negative solution, a contradiction. Hence, *N*_2_^−^ = *∅*.

*(IV) x* ∈ *N*_*l*_^−^ *on *[*t*_1_, *∞*)_*𝕋*_, *l* = 4,6,…, *n* − 1. In this case, we have that, for *t* ∈ [*t*_1_, *∞*)_*𝕋*_,

(55) x1(t)>0,  z(t)<0,  x2(t)<0,…,xl(t)<0,xl+1(t)>0,  xl+2(t)<0,…,xn(t)>0.

Integrating the first equation of (1) from *s* to *t*, we get that, for *s* ≤ *t* and *s*, *t* ∈ [*t*_1_, *∞*)_*𝕋*_,

(56) $$\begin{array}{c} z\left(t\right)\leq \int_{s}^{t}p_{1}\left(u_{1}\right)x_{2}\left(u_{1}\right)\Delta u_{1}. \end{array}$$

Integrating the 2th,…, (*l* − 1)th equation of (1), we obtain, for *t* ≥ *u*_1_ ≥ *u*_2_ ≥ ⋯≥*u*_*l*−2_ ≥ *s* and *s*, *u*_*k*_  (1 ≤ *k* ≤ *l* − 2), *t* ∈ [*t*_1_, *∞*)_*𝕋*_, that

(57) $$\begin{array}{c} x_{k}\left(u_{k-1}\right)\leq \int_{s}^{u_{k-1}}p_{k}\left(u_{k}\right)x_{k+1}\left(u_{k}\right)\Delta u_{k},\;2\leq k\leq l-1. \end{array}$$

Integrating the *l*th,…, (*n* − 1)th equation of (1), we obtain, for *t* ≥ *u*_*n*−1_ ≥ ⋯≥*u*_*l*−1_ ≥ *s* and *s*, *u*_*k*_  (*l* − 1 ≤ *k* ≤ *n* − 1), *t* ∈ [*t*_1_, *∞*)_*𝕋*_, that

(58) (−1)kxk(uk−1)≤(−1)k+1∫uk−1tpk(uk)xk+1(uk)Δuk,l≤k≤n−1.

Combining *n* − 1 inequalities above, we obtain that, for *t* ≥ *s* and *s*, *t* ∈ [*t*_1_, *∞*)_*𝕋*_,

(59) z(t)≤−∫stp1(u1)∫su1p2(u2)⋯∫sul−2pl−1(ul−1) ×∫ul−1tpl(ul)⋯ ×∫un−2tpn−1(un−1)xn(un−1)Δun−1,…,Δu1=−Il−1(t,s;p1,…,pl−2,pl−1(∗)×Jn−l(t,∗;pn−1xn,pn−2,…,pl)).

Note that (52) holds if *x*_1_(*t*) > 0, *z*(*t*) < 0, *x*_2_(*t*) < 0, and *x*_*n*_(*t*) > 0 for *t* ∈ [*t*_1_, *∞*)_*𝕋*_. So we have that, for *t* ≥ *u*_*n*−1_ ≥ *s* and *s*, *u*_*n*−1_, *t* ∈ [*t*_1_, *∞*)_*𝕋*_,

(60) $$\begin{array}{c} -x_{n}\left(u_{n-1}\right)<-K\left[\int_{u_{n-1}}^{t}\frac{p_{n}\left(u_{n}\right)}{a\left(g^{-1}\left(h\left(u_{n}\right)\right)\right)}\Delta u_{n}\right]z\left(g^{-1}\left(h\left(s\right)\right)\right). \end{array}$$

Putting *s* = *h* ^−1^(*g*(*t*))∈[*t*_1_, *∞*)_*𝕋*_, then

(61) $$\begin{array}{c} -x_{n}\left(u_{n-1}\right)<-K\left[\int_{u_{n-1}}^{t}\frac{p_{n}\left(u_{n}\right)}{a\left(g^{-1}\left(h\left(u_{n}\right)\right)\right)}\Delta u_{n}\right]z\left(t\right). \end{array}$$

Substituting (61) to (59), we have, on [*t*_2_, *∞*)_*𝕋*_ for some sufficiently large *t*_2_ ∈ *𝕋*, that

(62) z(t)≤[KIl−1(t,h−1(g(t));p1,…,pl−2,pl−1(∗)×Jn−l(t,∗;pn−1xn,pn−2,…,pl))]z(t).

So, for *t* ∈ [*t*_2_, *∞*)_*𝕋*_

(63) KIl−1(t,h−1(g(t));p1,…,pl−2,pl−1(∗)×Jn−l(t,∗;pn−1xn,pn−2,…,pl))≤1.

This contradicts (39) and hence, *N*_4_^−^ ∪ *N*_6_^−^ ∪ ⋯∪*N*_*n*−1_^−^ = *∅*.

*(V) x* ∈ *N*_*n*_^−^ *on *[*t*_1_, *∞*)_*𝕋*_. In this case, we have that, for *t* ∈ [*t*_1_, *∞*)_*𝕋*_,

(64) x1(t)>0,  z(t)<0,  x2(t)<0,  x3(t)<0,…,xn(t)<0.

Similarly as in the case (IV) of the proof of this theorem, we can get, for some sufficiently large *t*_2_ ∈ [*t*_1_, *∞*)_*𝕋*_, that

(65) $$\begin{array}{c} z\left(t\right)\leq KI_{n}\left(t,s;p_{1},\ldots ,p_{n-1},\frac{p_{n}z\left(g^{-1}\left(h\right)\right)}{a\left(g^{-1}\left(h\right)\right)}\right),\;t\in {\left[t_{2},\infty \right)}_{𝕋}. \end{array}$$

Using the monotonicity of *z*(*g* ^−1^(*h*(*t*))), we have, for *t* ∈ [*t*_2_, *∞*)_*𝕋*_, that

(66) z(t)≤[KIn(t,s;p1,…,pn−1,pna(g−1(h)))]z(t),t∈[t2,∞)𝕋.

So, for *t* ∈ [*t*_2_, *∞*)_*𝕋*_

(67) $$\begin{array}{c} KI_{n}\left(t,s;p_{1},\ldots ,p_{n-1},\frac{p_{n}}{a\left(g^{-1}\left(h\right)\right)}\right)\leq 1, \end{array}$$

which gives a contradiction with (40). Thus *N*_*n*_^−^ = *∅*. The proof is complete.

**Theorem**

Assume that *n* is even, *ι* = 1 in the system (1) and conditions (35)–(39) hold. Then, for every nonoscillatory solution *x* ∈ *W* to (1), we have lim⁡_*t*→*∞*_ *x*_*i*_(*t*) = 0, *i* = 1,2,…, *n*.

**Proof**

Let *x* ∈ *W* be a nonoscillatory solution to (1) and the expression (27) holds. Without loss of generality, we may assume that *x*_1_(*t*) > 0 for all *t* ∈ [*t*_1_, *∞*)_*𝕋*_ ⊂ [*t*_0_, *∞*)_*𝕋*_. We consider the following five cases.

*(I) x* ∈ *N*_1_^+^ *on *[*t*_1_, *∞*)_*𝕋*_. Analogically as the case (I) in the proof of Theorem 12, we can show that lim⁡_*t*→*∞*_ *x*_*i*_(*t*) = 0, *i* = 1,2,…, *n*.

*(II) x* ∈ *N*_3_^+^ ∪ *N*_5_^+^ ∪ ⋯∪*N*_*n*−1_^+^ ∪ *N*_*n*_^+^ *on *[*t*_1_, *∞*)_*𝕋*_. Also analogically as in the proof of Theorem 12(II), we can prove that *N*_3_^+^ ∪ *N*_5_^+^ ∪ ⋯∪*N*_*n*−1_^+^ ∪ *N*_*n*_^+^ = *∅*;

*(III) x* ∈ *N*_2_^−^ *on *[*t*_1_, *∞*)_*𝕋*_. In this case, we have

(68) x1(t)>,  z(t)<0,  x2(t)<0,  x3(t)>0,…,xn(t)<0,t∈[t1,∞)𝕋.

We continue analogically as (III) in the proof of Theorem 12. We can get, for *s* ≥ *t*  and  *s*, *t* ∈ [*t*_1_, *∞*)_*𝕋*_, that

(69) x2(t)≤−∫tsp2(u2)∫u2sp3(u3)⋯∫un−2spn−1(un−1) ×∫un−1sxnΔ(un)Δun⋯Δu2=−Jn−1(s,t;xΔ,pn−1,…,p2).

From (1), (b), (d), and (68), we have that, for *s* ≥ *u*_*n*_ ≥ *t* and *s*, *u*_*n*_, *t* ∈ [*t*_1_, *∞*)_*𝕋*_,

(70) $$\begin{array}{c} -x_{n}^{\Delta }\left(u_{n}\right)\leq -Kp_{n}\left(u_{n}\right)x_{1}\left(h\left(u_{n}\right)\right). \end{array}$$

We substitute (70) with (69), we have that, for *s* ≥ *u*_*n*_ ≥ *t* and *s*, *u*_*n*_, *t* ∈ [*t*_1_, *∞*)_*𝕋*_,

(71) $$\begin{array}{c} x_{2}\left(t\right)\leq KJ_{n-1}\left(s,t;-p_{n}x_{1}\left(h\right),p_{n-1},\ldots ,p_{2}\right). \end{array}$$

By the same way as in the proof of Theorem 12(III), we can obtain that *N*_2_^−^ = *∅*.

*(IV) x* ∈ *N*_*l*_^−^ *on *[*t*_1_, *∞*)_*𝕋*_, *l* = 4,6,…, *n* − 2. In this case, we have that, for *t* ∈ [*t*_1_, *∞*)_*𝕋*_,

(72) x1(t)>0,  z(t)<0,  x2(t)<0,…,xl(t)<0,  xl+1(t)>0,  xl+2(t)<0,…,xn(t)<0.

By the same way as in the proof of Theorem 12(IV), we can obtain, for *t* ≥ *s* and *s*, *t* ∈ [*t*_1_, *∞*)_*𝕋*_, that

(73) z(t)≤Il−1(t,s;p1,…,pl−2,pl−1(∗)×Jn−l(t,∗;pn−1xn,pn−2,…,pl)).

From (1), (b), (d) and (72), we have, for *s* ≥ *u*_*n*−1_ ≥ *t* and *s*, *u*_*n*−1_, *t* ∈ [*t*_1_, *∞*)_*𝕋*_, that

(74) $$\begin{array}{c} x_{n}\left(u_{n-1}\right)\leq -K\int_{u_{n-1}}^{s}p_{n}\left(u_{n}\right)x_{1}\left(h\left(u_{n}\right)\right)\Delta u_{n}. \end{array}$$

Therefore, it holds that, for *t* ≥ *s* and *s*, *t* ∈ [*t*_1_, *∞*)_*𝕋*_,

(75) z(t)≤−KIl−1(t,s;p1,…,pl−2,pl−1(∗)×Jn−l+1(t,∗;pnx1(h),pn−1,…,pl)).

Similarly as in the proof of Theorem 12(IV), we can prove that *N*_4_^−^ ∪ *N*_6_^−^ ∪ ⋯∪*N*_*n*−2_^−^ = *∅*.

*(V) x* ∈ *N*_*n*_^−^ *on *[*t*_1_, *∞*)_*𝕋*_. Similarly as in the proof of the case (IV) of this theorem, we can get, for *t* ≥ *s* and *s*, *t* ∈ [*t*_1_, *∞*)_*𝕋*_, that

(76) $$\begin{array}{c} z\left(t\right)\leq -KI_{n-1}\left(t,s;p_{1},\ldots ,p_{n-2},p_{n-1}\left(*\right)\times J_{1}\left(t,*;p_{n}x_{1}\left(h\right)\right)\right). \end{array}$$

And at the end to contradict to (39) for *l* = *n*. Thus *N*_*n*_^−^ = *∅*. This completes the proof.

**Theorem 14**

Let *n* be odd, *ι* = 1 in the system (1). If (37) the following statements hold:

(1) there is a constant *λ* such that, for any *t* ∈ [*t*_0_, *∞*)_*𝕋*_,
  (77) $$\begin{array}{c} 0<a\left(t\right)\leq \lambda <1; \end{array}$$
(2) for any *t* ∈ [*t*_0_, *∞*)_*𝕋*_,
  (78) $$\begin{array}{c} g\left(t\right)<t<h\left(t\right); \end{array}$$
(3) the function *α*(*t*) in (37) satisfies that
  (79) $$\begin{array}{c} \underset{t\to \infty }{\mathrm{limsup}}\int_{\sigma (t)}^{h(t)}Kp_{1}\left(v\right)J_{n-1}\left(\alpha \left(v\right),v;p_{n},p_{n-1},\ldots ,p_{2}\right)\Delta v>\frac{1}{e}; \end{array}$$
(4) for each even *l* with 4 ≤ *l* ≤ *n*,
  (80) limsup⁡t→∞KIl−1(t,h−1(t);p1,…,pl−2,pl−1(∗)×Jn−l+1(t,(∗);pn,…,pl))>1;
(5) (81) $$\begin{array}{c} \underset{t\to \infty }{\mathrm{limsup}}KI_{n}\left(t,h^{-1}\left(t\right);p_{1},p_{2},\ldots ,p_{n}\right)>1. \end{array}$$

Then, for every nonoscillatory solution *x* ∈ *W* to* (1)*, lim⁡_*t*→*∞*_ *x*_*i*_(*t*) = 0, *i* = 1,2,…, *n*.

**Proof**

Let *x* ∈ *W* be a nonoscillatory solution to (1). Without loss of generality, we may assume that *x*_1_(*t*) > 0 for all *t* ∈ [*t*_1_, *∞*)_*𝕋*_ ⊂ [*t*_0_, *∞*)_*𝕋*_. By the expression (25) and Remark 9, we have *N* = *N*_2_^+^ ∪ *N*_4_^+^ ∪ ⋯∪*N*_*n*−1_^+^ ∪ *N*_*n*_^+^ ∪ *N*_1_^−^. We consider the following four cases.

*(I) x* ∈ *N*_2_^+^ *on *[*t*_1_, *∞*)_*𝕋*_. In this case, we have that

(82) x1(t)>0,  z(t)>0,  x2(t)>0,  x3(t)<0,…,xn(t)<0,t∈[t1,∞)𝕋.

Integrating the second equation of (1) from *t* to *s*, we obtain that, for *s* ≥ *t* and *s*, *t* ∈ [*t*_1_, *∞*)_*𝕋*_,

(83) $$\begin{array}{c} x_{2}\left(t\right)\geq -\int_{t}^{s}p_{2}\left(u_{2}\right)x_{3}\left(u_{2}\right)\Delta u_{2}. \end{array}$$

Integrating the *k*th equation of (1) from *u*_*k*−1_ to *s*, we get, for *s* ≥ *u*_*k*−1_ ≥ *t* and *s*, *u*_*k*−1_, *t* ∈ [*t*_1_, *∞*)_*𝕋*_, that

(84) (−1)kxk(uk−1)≥(−1)k+1∫uk−1spk(uk)xk+1(uk)Δuk,3≤k≤k−1,−xn(un−1)≥∫un−1sxnΔ(un)Δun.

Combining *n* − 1 inequalities above, we obtain that, for *s* ≥ *t* and *s*, *t* ∈ [*t*_1_, *∞*)_*𝕋*_,

(85) x2(t)≥∫tsp2(u2)∫u2sp3(u3)⋯∫un−2spn−1(un−1) ×∫un−1sxnΔ(un)Δun⋯Δu2=Jn−1(s,t;xnΔ,pn−1,…,p2).

From (1), (b), (d), and (44), we have that, for *s* ≥ *u*_*n*_ ≥ *t* and *s*, *u*_*n*_, *t* ∈ [*t*_1_, *∞*)_*𝕋*_,

(86) $$\begin{array}{c} x_{n}^{\Delta }\left(u_{n}\right)\geq Kp_{n}\left(u_{n}\right)x_{1}\left(h\left(u_{n}\right)\right). \end{array}$$

Substitute (86) with (71), and notice that the inequality *x*_1_(*h*(*t*)) ≥ *z*(*h*(*t*)) holds for any *t* ∈ [*t*_1_, *∞*)_*𝕋*_. Then we have that, for *s* ≥ *t* and *s*, *t* ∈ [*t*_1_, *∞*)_*𝕋*_,

(87) x2(t)≥KJn−1(s,t;pnz(h),pn−1,…,p2)≥KJn−1(s,t;pn,pn−1,…,p2)z(h(t)).

Multiplying the inequality above by *p*_1_(*t*) and putting *s* = *α*(*t*), we get that

(88) zΔ(t)−[Kp1(t)Jn−1(α(t),t;pn,pn−1,…,p2)]z(h(t))≥0,t∈[t1,∞)𝕋.

By Lemma 6, the inequality above has no eventually positive solution, a contradiction. Hence, *N*_2_^+^ = *∅*.

*(II) x* ∈ *N*_*l*_^+^ *on *[*t*_1_, *∞*)_*𝕋*_, *l* = 4,6,…, *n* − 1. In this case, we have that, for any *t* ∈ [*t*_1_, *∞*)_*𝕋*_,

(89) x1(t)>0,  z(t)>0,  x2(t)>0,…,xl(t)>0,xl+1(t)<0,  xl+2(t)>0,…,xn(t)<0.

Integrating the first equation of (1) over [*s*, *t*]_*𝕋*_, we get that

(90) $$\begin{array}{c} z\left(t\right)\geq \int_{s}^{t}p_{1}\left(u_{1}\right)\Delta u_{1},\;s\leq t,\;s,t\in {\left[t_{1},\infty \right)}_{𝕋}. \end{array}$$

Similarly, by integrating the 2th,…, (*l* − 1)th equations of (1) and the obtained inequalities institute to (90), we get, for *s* ≤ *t*  and  *s*, *t* ∈ [*t*_1_, *∞*)_*𝕋*_, that

(91) z(t)≥∫stp1(u1)∫su1p2(u2)⋯ ×∫sul−2pl−1(ul−1)xl(ul−1)Δul−1⋯Δu1=Il−1(t,s;p1,…,pl−2,pl−1xl).

Integrating the *l*th,…, *n*th equation of (1), we obtain, for *t* ≥ *u*_*n*−1_ ≥ ⋯≥*u*_*l*−1_ ≥ *s* ≥ *t*_1_ and *s*, *u*_*k*_  (*l* − 1 ≤ *k* ≤ *n* − 1), *t* ∈ [*t*_1_, *∞*)_*𝕋*_, that

(92) (−1)kxk(uk−1)≥(−1)k+1∫uk−1tpk(uk)xk+1(uk)Δuk,l≤k≤n−1,−xn(un−1)≥∫un−1txnΔ(un)Δun.

Combining *n* − *l* + 1 inequalities above and (91), we obtain that, for *t* ≥ *s* and *s*, *t* ∈ [*t*_1_, *∞*)_*𝕋*_,

(93) z(t)≥∫stp1(u1)∫su1p2(u2)⋯∫sul−2pl−1(ul−1) ×∫ul−1tpl(ul)⋯∫un−2tpn−1(un−1)xn(un−1) ×∫un−1txnΔ(un)Δun−1⋯Δu1=Il−1(t,s;p1,…,pl−2,pl−1(∗)×Jn−l+1(t,∗;xnΔ,pn−1,…,pl)).

Note that (86) and the inequality *x*_1_(*h*(*t*)) ≥ *z*(*h*(*t*)) hold. So we have that, for *t* ≥ *s* and *s*, *t* ∈ [*t*_1_, *∞*)_*𝕋*_,

(94) z(t)≥KIl−1(t,s;p1,…,pl−2,pl−1(∗)×Jn−l+1(t,∗;pnz(h),pn−1,…,pl)).

Putting *s* = *h* ^−1^(*t*)∈[*t*_1_, *∞*)_*𝕋*_, using the monotonicity of *z*(*h*(*t*)), then we have, for some sufficiently large *t*_2_ ∈ [*t*_1_, *∞*)_*𝕋*_, that

(95) z(t)≥[KIl−1(t,h−1(t);p1,…,pl−2,pl−1(∗)     ×Jn−l+1(t,∗;pn,…,pl))]z(t),t∈[t2,∞)𝕋.

So, for *t* ∈ [*t*_2_, *∞*)_*𝕋*_,

(96) KIl−1(t,s;p1,…,pl−2,pl−1(∗)×Jn−l(t,∗;pn−1xn,pn−2,…,pl))≤1.

This contradicts (80) and hence, *N*_4_^+^ ∪ *N*_6_^+^ ∪ ⋯∪*N*_*n*−1_^+^ = *∅*.

*(III) x* ∈ *N*_*n*_^+^ *on *[*t*_1_, *∞*)_*𝕋*_. In this case, we have that, for *t* ∈ [*t*_1_, *∞*)_*𝕋*_,

(97) x1(t)>0,  z(t)>0,  x2(t)>0,  x3(t)>0,…,xn(t)>0.

Analogically as derived at part (II) of the proof of this theorem, we have that

(98) $$\begin{array}{c} z\left(t\right)\geq I_{n}\left(t,s;p_{1},\ldots ,p_{n-1},x_{n}^{\Delta }\right),\;t\geq s,\;s,t\in {\left[t_{1},\infty \right)}_{𝕋}. \end{array}$$

And putting *s* = *h* ^−1^(*t*), we can obtain that

(99) $$\begin{array}{c} KI_{n}\left(t,h^{-1}\left(t\right);p_{1},\ldots ,p_{n}\right)\leq 1,\;t\in {\left[t_{1},\infty \right)}_{𝕋}, \end{array}$$

which give a contradiction with (81), and so *N*_*n*_^+^ = *∅*.

* (IV) x* ∈ *N*_1_^−^ *on *[*t*_1_, *∞*)_*𝕋*_. In this case, we have that

(100) x1(t)>0,  z(t)<0,  x2(t)>0,  x3(t)<0,…,xn(t)<0,t∈[t1,∞)𝕋.

By Lemma 5, lim⁡_*t*→*∞*_ *x*_1_(*t*) = 0. From (100), we have lim⁡_*t*→*∞*_ | *z*(*t*)| = *L*_1_ < +*∞*. Then it follows, by Lemma 10, that lim⁡_*t*→*∞*_ *x*_*i*_(*t*) = 0, *i* = 2,3,…, *n*. The proof is complete.

Similarly to Theorem 14, we can prove the following theorem.

**Theorem 15**

Assume that *n* is even, *ι* = −1 in the system (1) and conditions (77)–(80) hold. Then, for every nonoscillatory solution *x* ∈ *W* to (1), we have lim⁡_*t*→*∞*_ *x*_*i*_(*t*) = 0, *i* = 1,2,…, *n*.

**Example**

We consider a system on the time scale $T=\bar{q^{\mathbb{Z}}}$. In order to simplify calculations, we may suppose that *q* = 2. Let *a*(*t*) = 8, *g*(*t*) = 16*t*, *h*(*t*) = 512*t*, *p*_1_(*t*) = (1/4)*t*, *p*_2_(*t*) = (7/8)*t*, *p*_3_(*t*) = (31/32)*t*, *p*_4_(*t*) = (127/128)*t*, *p*_5_(*t*) = 511/*t* ^9^, *f*(*y*) = *y*, *K* = 1, *α*(*t*) = 2*t*, *ι* = −1, and *n* = 5; that is to say, the system has the following form:

(101) [x1(t)−8x1(16t)]Δ=14tx2(t),x2Δ(t)=78tx3(t),x3Δ(t)=3132tx4(t),x4Δ(t)=127128tx5(t),x5Δ(t)=−511t9x1(512t), t∈𝕋.

We calculate the conditions of Theorem 12 as follows:

(102) ∫2t32t14v∫v2v78x1∫x12v3132x2∫x22v127128x3  ×∫x32v5118x49Δx4Δx3Δx2Δx1Δv≈234.05>1e,∫2−5tt14x1∫2−5tx178x2∫2−5tx23132x3∫x3t127128x4  ×∫x4t5118x59Δx5Δx4Δx3Δx2Δx1≈332233.05>1,∫2−5tt14x1∫2−5tx178x2∫2−5tx23132x3∫2−5tx3127128x4  ×∫2−5tx45118x59Δx5Δx4Δx3Δx2Δx1≈3431.69>1.

It follows, from Theorem 12, that for every nonoscillatory solution *x* ∈ *W* to (101), lim⁡_*t*→*∞*_ *x*_*i*_(*t*) = 0, *i* = 1,2,…, 5. In fact, functions *x*_1_(*t*) = 1/*t*, *x*_2_(*t*) = −1/*t* ^3^, *x*_3_(*t*) = 1/*t* ^5^, *x*_4_(*t*) = −1/*t* ^7^, and *x*_5_(*t*) = 1/*t* ^9^ are particular components of such a kind of solutions.

**Example 17**

We also consider a system on the time scale $T=\bar{q^{\mathbb{Z}}}$ and let *q* = 2. Let *a*(*t*) = 2, *g*(*t*) = 4*t*, *h*(*t*) = 128*t*, *p*_1_(*t*) = (1/8)*t*, *p*_2_(*t*) = (1/4)*t*, *p*_3_(*t*) = (7/32)*t*, *p*_4_(*t*) = 31/*t* ^7^, *f*(*y*) = 127*y*, *K* = 127, *α*(*t*) = 16*t*, *ι* = 1, and *n* = 4; that is to say, the system has the following form:

(103) [x1(t)−2x1(4t)]Δ=18tx2(t),x2Δ(t)=14tx3(t),x3Δ(t)=732tx4(t),x4Δ(t)=31t7·127·x1(128t), t∈𝕋.

We calculate the conditions of Theorem 13 as follows:

(104) ∫2t32t12723v∫v24v14x1∫x124v732x2  ×∫x224v312x37Δx3Δx2Δx1Δv≈77.39>1e,127∫2−5tt18x1∫2−5tx114x2∫2−5tx2732x3  ×∫x3t312x47Δx4Δx3Δx2Δx1≈331582.88>1.

It follows, from Theorem 13, that for every nonoscillatory solution *x* ∈ *W* to (103), lim⁡_*t*→*∞*_ *x*_*i*_(*t*) = 0, *i* = 1,2,…, 4. In fact, functions *x*_1_(*t*) = 1/*t*, *x*_2_(*t*) = −2/*t* ^3^, *x*_3_(*t*) = 7/*t* ^5^, and *x*_4_(*t*) = −31/*t* ^7^ are particular components of such a kind of solutions.
